# Celiac disease and complement activation in response to *Streptococcus pneumoniae*

**DOI:** 10.1007/s00431-019-03490-w

**Published:** 2019-11-05

**Authors:** Anna Röckert Tjernberg, Hanna Woksepp, Kerstin Sandholm, Marcus Johansson, Charlotte Dahle, Jonas F Ludvigsson, Jonas Bonnedahl, Per Nilsson, Kristina Nilsson Ekdahl

**Affiliations:** 1 Department of Pediatrics, Kalmar County Hospital, SE-391 85 Kalmar, Sweden; 2 School of Medical Sciences, Örebro University, SE-701 82 Örebro, Sweden; 3 Research section, Department of Development and Public Health, Kalmar County Hospital, SE-391 85 Kalmar, Sweden; 4 Linnaeus Center for Biomaterials Chemistry, Linnaeus University, SE-391 82 Kalmar, Sweden; 5 Department of Clinical Microbiology, Kalmar County Hospital, SE-391 85 Kalmar, Sweden; 6 Department of Clinical and Experimental Medicine, Linköping University, SE-581 85 Linköping, Sweden; 7 Department of Clinical Immunology and Transfusion Medicine, Linköping University Hospital, SE-581 85 Linköping, Sweden; 8 Department of Medical Epidemiology and Biostatistics, Karolinska Institutet, SE-171 77 Stockholm, Sweden; 9 Department of Pediatrics, Örebro University Hospital, SE-701 85 Örebro, Sweden; 10 Department of Immunology, Oslo University Hospital, University of Oslo, 0424 Oslo, Norway; 11 Department of Immunology, Genetics and Pathology, Uppsala University, SE-751 85 Uppsala, Sweden

**Keywords:** Coeliac, Pneumococcal, Infection, Innate immunity, MBL

## Abstract

Individuals with celiac disease (CD) are at increased risk of invasive pneumococcal disease (IPD). The aim of this study was to explore whether the complement response to *Streptococcus pneumoniae* differed according to CD status, and could serve as an explanation for the excess risk of IPD in CD. Twenty-two children with CD and 18 controls, born 1999–2008, were included at Kalmar County Hospital, Sweden. The degree of complement activation was evaluated by comparing levels of activation products C3a and sC5b-9 in plasma incubated for 30 min with *Streptococcus pneumoniae* and in non-incubated plasma. Complement analyses were performed with enzyme-linked immunosorbent assay (ELISA). Pneumococcal stimulation caused a statistically significant increase in C3a as well as sC5b-9 in both children with CD and controls but there was no difference in response between the groups. After incubation, C3a increased on average 4.6 times and sC5b-9 22 times in both the CD and the control group (*p* = 0.497 and *p* = 0.724 respectively).

*Conclusion*: Complement response to *Streptococcus pneumoniae* seems to be similar in children with and without CD and is thus unlikely to contribute to the increased susceptibility to invasive pneumococcal disease in CD.

**Table:** 

**What is Known:** • *An excess risk of pneumococcal infections has been demonstrated in individuals with celiac disease.* *• Infectious complications can depend on hyposplenism but alternative mechanisms are sparsely examined.*	
**What is New:** *• Complement activation in response to Streptococcus pneumoniae was examined in children with and without celiac disease but no differences could be demonstrated.*

**What is Known:**

• *An excess risk of pneumococcal infections has been demonstrated in individuals with celiac disease.*

*• Infectious complications can depend on hyposplenism but alternative mechanisms are sparsely examined.*

**What is New:**

*• Complement activation in response to Streptococcus pneumoniae was examined in children with and without celiac disease but no differences could be demonstrated.*

## Introduction

Celiac disease (CD) is a chronic enteropathy affecting about 1% of the population worldwide [15]. The last decade, severe infections as complications of CD have received increased attention [14, 29]. In particular, pneumococcal infections have attracted interest and several studies, including our own [25], have shown an increased risk of invasive pneumococcal disease (IPD) in CD (HRs = 1.4–2.5) [13, 25, 28]. Since IPD is a potentially life-threatening condition, these findings have resulted in changed guidelines for CD management [2]. In the UK, pneumococcal immunization is now recommended to individuals with CD [16]. CD is an autoimmune disease and is considered to be one of the most common disorders associated with splenic hypofunction and atrophy [8], even though the most recent estimates are slightly lower than the first reports [6]. The spleen is crucial for the defense against encapsulated bacteria and the elevated risk of IPD in CD has been attributed to this possible hyposplenism [7]. However, an impaired splenic function is rarely seen in children [3] and moreover, it is believed to improve after introduction of a gluten-free diet (GFD) [4] whereas the excess risk of IPD seems to persist beyond one year of follow-up [13, 25, 28]. In the light of this, we deemed that alternative explanations for this increased susceptibility deserved further attention.

The innate immunity is the first line of defense against pathogens, in which the complement system plays an important role [24]. The complement system is a complex network of proteins acting in a cascade-like manner [24]. Its key functions are the opsonization of the surface of the pathogen, activation and recruitment of circulating and tissue-resident inflammatory cells, and the direct killing of the bacteria via formation of the membrane attack complex (MAC) [23, 24]. The complement system can be activated through different pathways (classical, lectin, or alternative) [23, 24]. Data from mice models have shown that complement protection against pneumococci is mainly mediated through the classical pathway which is activated by antigen-bound antibodies and by acute phase pentraxins. The alternative pathway is in general also activated but the response seems, in comparison, weaker. The lectin pathway, also important in the defense against pneumococci, is activated by binding of mannan-binding lectin (MBL) and ficolins to microbes [1, 23, 24]. Complement deficiencies, including MBL deficiency, lead to an increased risk of pneumococcal infections or adverse outcome in IPD [24]. The knowledge of the complement system’s role in CD is limited. Some older studies have shown reduced levels of C3 and C4 [17, 27]; however, none of them has measured the levels of complement activation markers in response to a pathogen.

The aim of this study was to investigate whether the complement response to *Streptococcus pneumoniae* differed between young individuals with and without CD.

## Material and methods

### Study participants

All study participants were included at the Pediatric Clinic at Kalmar County Hospital, Sweden. The participants were born between 1999 and 2008. Since plasma levels of complement components as well as complement activation are independent of age and sex in this age category [5, 19], no matching was performed. We chose to include individuals born 2008 and earlier since pneumococcal immunization was included in the Swedish national vaccine program for children in 2009 [26].

### CD

Individuals with CD, born 1999–2008, were identified through computerized medical records and invited to participate in the study through a study-specific letter. Patients with an upcoming visit in near-time were invited first. Patients with additional autoimmune diseases or ongoing infection were excluded.

### Controls

Individuals, born 1999–2008, visiting the Pediatric Clinic for other reasons than CD were invited to participate as controls. Individuals with autoimmune diseases or ongoing infection were excluded.

## Methods

### Clinical data

All study participants filled out a questionnaire about diet, medication, autoimmune diseases, previous pneumonias/meningitis, pneumococcal vaccine, and known splenic affection. In addition, medical records were reviewed when there were uncertainties.

### Blood sampling and preparation of plasma

Blood samples for analyses of complement activation products (C3a and sC5b-9), C3, MBL, pneumococcal serology, and IgA antibodies against tissue transglutaminase (tTG) were collected from all study participants. Plasma-EDTA for complement analyses was centrifuged at 2500×*g* for 20 min and frozen at − 70 °C, within 4 h from sampling.

### Pneumococcal incubations in lepirudin plasma

Prior to pneumococcal stimulation, the plasma anticoagulant EDTA was removed to allow for complement activation. Samples were spinned through Bio-Spin P-6 gel columns (Bio-Rad Laboratories AB, Solna, Sweden), saturated with veronal-buffered saline and lepirudin 50 μg/mL (Refludan®, Celgene, Windsor, UK) as previously described [9]. For pneumococcal stimulation, *Streptococcus pneumoniae* (serogroup 23F) isolated from a patient suffering from invasive infection was chosen. The isolate was retrieved from the Department of Clinical Microbiology, Kalmar County Hospital, Sweden. Pneumococcal stimulation was carried out by mixing 20 μL of *S. pneumoniae*, 10^8^ CFU/mL in NaCl with 180 μL plasma. As control 20 μL 0.9% NaCl without bacteria was added. Incubation was done in polypropylene microtubes, 30 min in 37 water-bath. An additional control was included: 20 μL NaCl mixed with 180 μL plasma, without incubation. The reaction was stopped by adding 0.2 M EDTA (10 mM final concentration). Incubations were carried out as duplicates or triplicates, depending on sample volume. Samples were centrifuged at 4500×*g* for 5 min and 150 μL was frozen at − 80 °C prior to complement analysis.

### Analysis of complement activation (C3a and sC5b-9)

Complement activation was monitored as the generation of activation products C3a and sC5b-9 complexes measured in the plasma by employing enzyme-linked immunosorbent assay (ELISA) with antibodies specific for neo-epitopes in C3a and C9 respectively, as previously described [18, 20]. For statistical analyses, we used the median of the triplicates and duplicates. The concentration was presented in μg/mL.

### C3

Total C3 was measured by nephelometry (Beckman Coulter Immage 800, Bromma, Sweden) using Immunochemistry Diagnostic C3 (Beckman). Analyses were performed at the Department of Clinical Immunology and Transfusion Medicine, Uppsala, Sweden.

### Mannan-binding lectin

MBL was measured by sandwich ELISA using mouse monoclonal antibody (clone HYB 131-01) from Santa Cruz Biotechnology Inc. (Santa Cruz, CA). Analyses were performed at the Department of Clinical Immunology and Transfusion Medicine, Uppsala, Sweden.

### Pneumococcal serotype-specific IgG measurement (pneumococcal serology)

IgG antibodies against pneumococcal serotypes 19F, 23F, and 6B were quantified using ELISA meeting World Health Organization standard [21]. The analyses were performed at the Department of Clinical Immunology and Transfusion Medicine, Lund, Sweden.

### IgA antibodies against tissue transglutaminase

IgA antibodies against tissue transglutaminase were analyzed with Thermo Fisher Scientific Phadia 250 (Thermo Fisher Scientific, Uppsala, Sweden) at the accredited hospital laboratory at Kalmar County Hospital. The method includes a screening for detecting IgA deficiency (EliA™ Celikey® IgA on Phadia 250, Thermo Fisher Scientific, Uppsala, Sweden). In case of a low response, samples are further analyzed on BN ProSpec (Siemens, Erlangen, Germany) to get an actual IgA level [12]. If IgA is ≤ 0.07 g/L, IgG antibodies against transglutaminase and IgG antibodies against deamidated gliadin peptide are measured.

## Statistics

Variables were examined with normality tests (Kolmogorov-Smirnov, Shapiro-Wilk, and plots). Non-parametric tests (Mann-Whitney *U* test) were used for group comparisons. In addition, parametric tests (independent sample *t* test) were used when variables were normally distributed. To statistically verify that pneumococcal stimulation caused complement activation, the Wilcoxon signed-rank test was used. Spearman’s rho was used for analyzing correlations.

Analyses and figures were performed using SPSS 24 (SPSS, Inc., Chicago, IL, USA) and GraphPad Prism version 7.0 (GraphPad Software, San Diego, CA). *p* values < 0.05 were considered statistically significant.

## Ethics

The study conforms to the ethical guidelines of the 1975 Declaration of Helsinki and was approved by the Ethical Review Board in Linköping, Sweden (2016/366/31). Written informed consent was obtained from all participants and their guardians.

## Results

### Characteristics of study participants

A total of 25 individuals with CD and 23 controls were included. However, during the process, 8 study participants had to be excluded: two individuals with CD and two controls due to bacterial contamination, one CD and one control due to too low bacterial concentration, and one control did, for unknown reason, not activate C3a and sC5b-9 (despite C3 level within normal range), in addition, plasma from one control was missing. Accordingly, the final study population consisted of 22 individuals with CD and 18 controls. The median age was 15.4 (10.2–17.1) years in the CD group and 11.5 (9.3–17.4) years in the control group. The majority of the study participants were female (CD 16/22 and controls 10/18). None of the participants had received pneumococcal vaccination. One CD patient claimed to have had more than one pneumonia but the diagnoses could not be verified. No recent (last month) pneumonias or previous meningitis were reported. No participant had a known affection of the spleen or reported use of medication that potentially could affect pneumococcal stimulation or complement activity. One of the CD patients had a suspected asthma diagnosis; otherwise, no comorbidities known to influence complement levels were reported or found when reviewing medical records. All but one of the CD patients received their diagnosis after small intestinal biopsy showing findings compatible with CD. The remaining patient was diagnosed without biopsy as proposed by the European Society for Pediatric Gastroenterology, Hepatology and Nutrition (ESPGHAN) guidelines from 2012 [10]. The mean duration of CD was 7.6 years (1.3–14.9). Out of the control individuals, 15/18 attended the hospital for undergoing magnetic resonance imaging. Four of the controls reported having a relative with CD (responding rate 17/18).

## Laboratory results

### Pneumococcal-induced complement activation

#### C3a

**Fig. 1:**
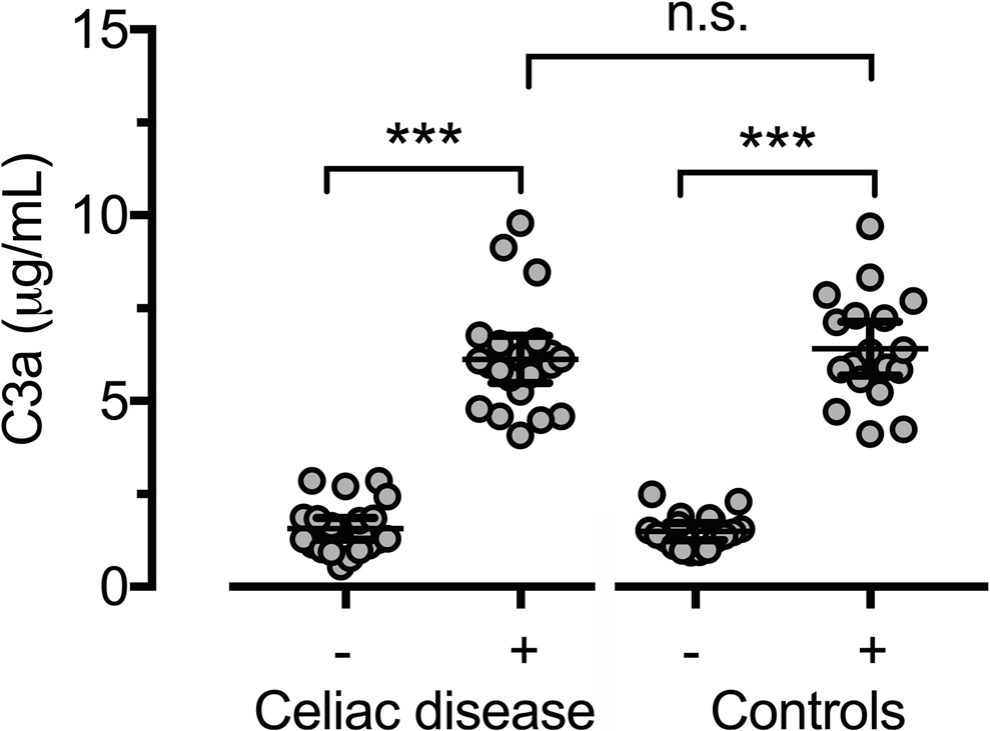
Distribution of C3a in individuals with CD and controls before and after pneumococcal stimulation. Bars representing mean and 95%CI. (−) Non-stimulated samples. (+) Stimulated samples

Pneumococcal stimulation did cause a statistically significant increase in C3a levels in both the CD (*p* < 0.001) and the control group (*p* < 0.001) but the levels did not differ between the groups (*p* = 0.497) (Fig. 1). After incubation, C3a increased on average 4.6 times in both the CD and the control group (CD: median 4.4; range 1.6–11.4 and controls: median 4.7; range 2.3–6.7). Additional statistical analyses comparing C3a concentrations in non-stimulated and stimulated samples showed no significant differences between the groups (*p* = 0.415 and *p* = 0.786 respectively).

#### C3 and C3a/C3 ratio

**Fig. 2:**
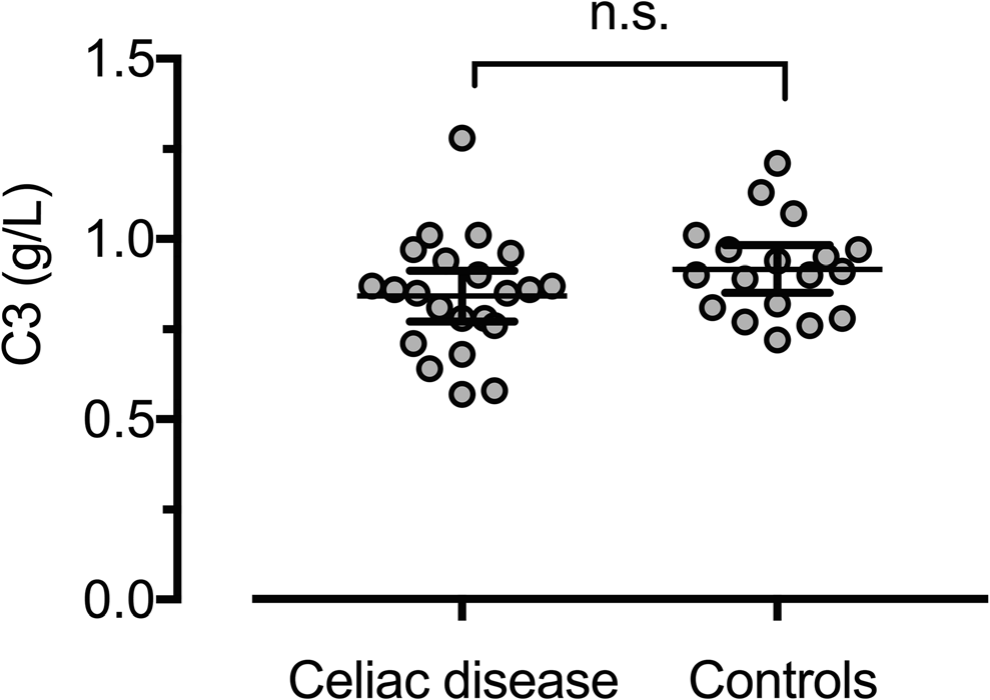
Distribution of C3 in individuals with CD and controls. Bars representing mean and 95%CI

**Fig. 3:**
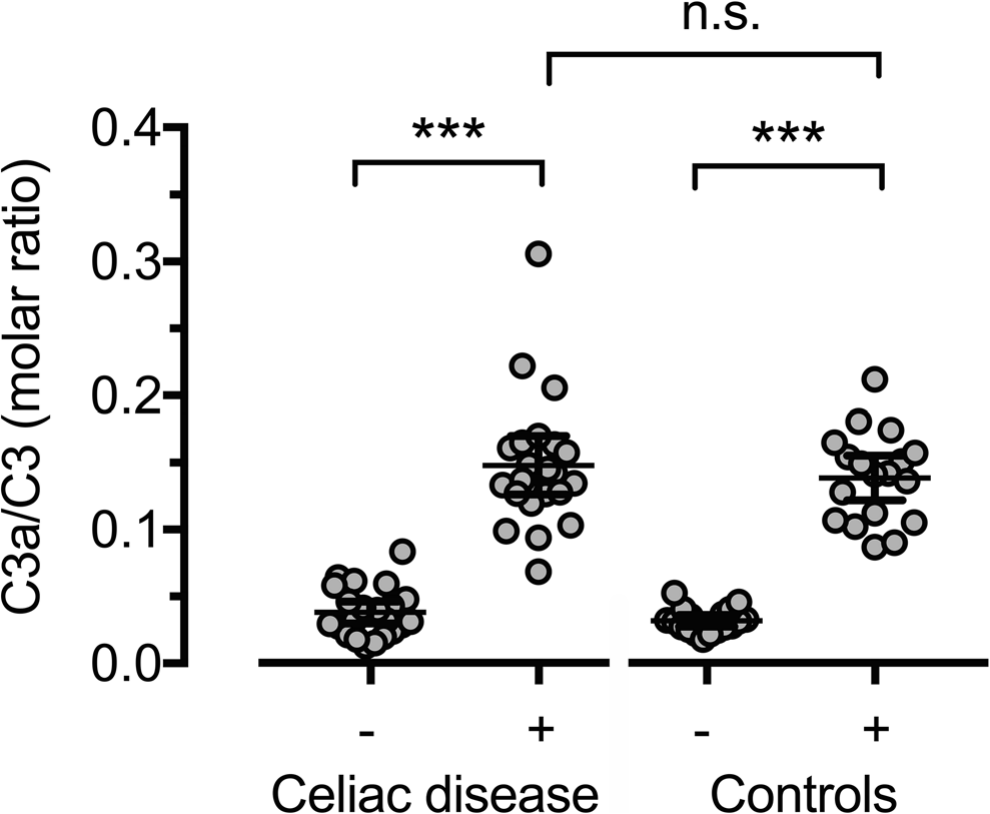
Distribution of C3a/C3 ratio in individuals with CD and controls before and after pneumococcal stimulation. Bars representing mean and 95%CI. (−) Non-stimulated samples. (+) Stimulated samples

Since C3a is dependent on the total C3 level, we also measured C3 concentrations. The C3 values were normally distributed and statistical testing showed no significant difference between the groups (*p* = 0.124) (Fig. 2). In addition, we examined the ratio between C3a and C3 since this is reported to be a more sensitive indicator than C3a alone [30] (Fig. 3). No statistically significant differences between the study groups could be found. Three CD patients had C3 levels below the reference. Excluding these from the statistical analyses did not affect the results.

#### sC5b-9

**Fig. 4:**
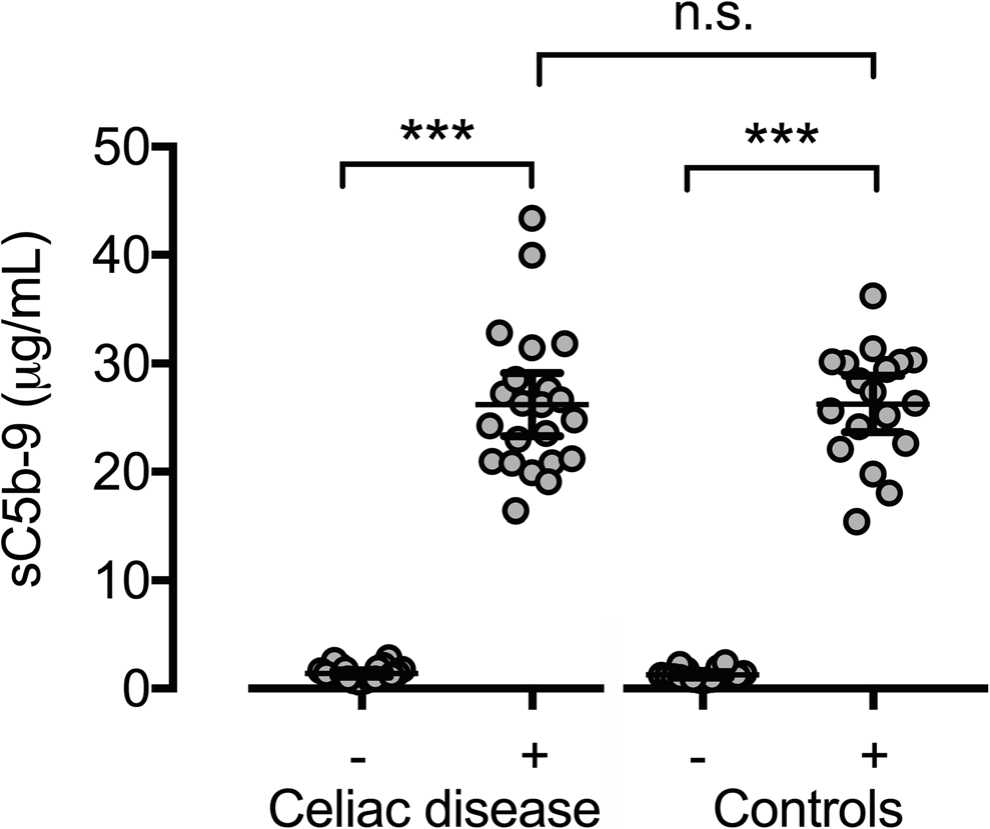
Distribution of sC5b-9 in individuals with CD and controls before and after pneumococcal stimulation. Bars representing mean and 95%CI. (−) Non-stimulated samples. (+) Stimulated samples

Pneumococcal stimulation was statistically significantly associated with an increase in sC5b-9 activity in both the CD (*p* < 0.001) and the control group (*p* < 0.001) but no significant difference was seen between the groups (*p* = 0.724) (Fig. 4). In the CD group, sC5b-9 increased on average 22 times (median 19; range 8–47), equal to the also 22-fold increase observed in the control group (median 20; range 5–39). Comparing the study groups with regard to sC5b-9 concentrations in non-stimulated and stimulated samples separately did not reveal any statistically significant differences (*p* = 0.881 and *p* = 0–664 respectively).

### Concentration of Streptococcus pneumoniae

The bacterial load was evaluated after each pneumococcal stimulation. CFU varied between 6.8 × 10^6^ and > 5 × 10^8^ (median 5 × 10^8^). Statistical testing showed no difference in distribution of bacterial concentration between the CD group and controls (*p* = 0.757).

### Mannan-binding lectin

The median concentration of MBL was 481.5 kU/L (7 to > 2000 kU/L) in the CD group and 492.5 kU/L in controls (102 to > 2000 kU/L). There was no statistically significant difference between the study groups (*p* = 0.508). One study participant (CD individual) had an MBL concentration below the reference level (7 kU/L [ref > 40 kU/L]). The results from the statistical analyses did not change when this participant was removed from the data set.

### Pneumococcal serology

The majority of the study participants showed signs of previous pneumococcal infection (IgG antibody level > 1 mg/L). In the CD group, the median IgG antibody concentration for pneumococcal serotype 19F was 3.10 (0.10 to 38.00) mg/L, for serotype 23F 2.00 (0.22 to > 26) mg/L and for serotype 6B 5.25 (0.29 to > 50) mg/L. The corresponding numbers for controls (*n* = 16, samples missing from two controls) were 4.25 (0.53 to 23) mg/L, 1.5 (0.34 to > 26) mg/L, and 3.50 (0.22 to > 50) mg/L respectively. Mann-Whitney *U* tests showed no statistically significant differences in distribution between the study groups in any of the serotypes (*p* = 0.535, *p* = 0.431, and *p* = 0.460 respectively). Antibody levels did not correlate to the degree of complement response in any of the serotypes.

### IgA antibodies against tissue transglutaminase

Two individuals in the CD group had a slightly elevated tTG (8 and 13 kIE/L respectively [ref < 7 kIE/L]). Excluding these individuals from statistical analyses did not change the results. None of the study participants had p-IgA < 0.07 g/L wherefore there was no need for assessment of alternative serological CD markers.

## Discussion

The first reports of severe pneumococcal infections in individuals with CD were published in the 1980s [22]. The last decade, the association between CD and IPD has been examined more thoroughly [13, 25, 28] and recent international CD guidelines recommend pneumococcal vaccine in CD [2, 16]. The complement system plays an important role in the immune system’s first line of defense against invading pathogens, including *Streptococcus pneumoniae* [23] but also against other encapsulated bacteria (e.g., meningococci and *Haemophilus Influenzae*). These latter pathogens have been very sparsely examined in CD although some experts argue that vaccine against these bacteria should be offered in addition to pneumococcal immunization. Even though complement deficiency is not uncommon in children with recurrent IPD [11], to our knowledge, ours is the first study to investigate whether alterations in the complement system are linked to the increased risk of IPD in CD. We used plasma samples from children (9–18 years old) with and without CD to measure complement activation, i.e., levels of complement activation markers C3a and sC5b-9, after in vitro stimulation with pneumococci. Even though pneumococci might resist the MAC (C5b-9) [23], we chose to measure the soluble form of this complex (sC5b-9) in addition to C3a since they are the complement cascade’s terminal products [24] and accordingly reflect the function of the entire complement system. A complement response was evident in all individuals after incubation with *S. pneumoniae*, but no differences in activation between the CD group and controls could be demonstrated.

Besides assessing the increased susceptibility to infections in CD from a totally new angle, one of the major strengths of the study was the availability of clinical data. All CD diagnoses were confirmed by reviewing relevant medical records. All CD cases but one were biopsy-verified. The absence of concomitant autoimmune diseases could also be confirmed. We also had access to vaccine data. The Swedish national vaccine register (Swevac) has a good coverage in the county where the study was performed. Likewise, medical records of control children were reviewed. This was done in addition to the questionnaire to confirm that the controls did not have any autoimmune diseases other than CD or other conditions that potentially could affect complement activity, thereby avoiding selection bias. Since the study did not include infants, we did not require age and sex matching between the study groups. The youngest patient in the study was 9 years old and by that age complement levels and response have reached adult levels [5, 19]. To further confirm this (and by that excluding selection bias in regard to sex and age), we analyzed total C3 and no difference was found according to CD status. The mean duration of CD was 7.6 years and all patients claimed to adhere to a GFD which makes ongoing intestinal inflammation unlikely. However, two CD patients had a slightly elevated IgA tTG. They had only been diagnosed with CD 1.3 and 1.8 years ago respectively and it is therefore possible that they had not yet acquired complete mucosal recovery. To examine if these patients somehow influenced our results, we also performed all statistical tests after excluding them from the analyses but this did not affect the results.

The laboratory work was performed in several steps. Prior to stimulation, pilot tests were performed to estimate bacterial load and incubation time needed to induce complement activation in vitro. Initially lower concentrations of pneumococci (100, 1000, and 10,000 CFU/mL) were evaluated but these failed to activate the complement and a concentration of 10^8^ CFU/mL was required. High bacterial loads are commonly required to activate complement in vitro. Even though this could be considered a limit, it is unlikely to affect group comparisons. Incubation times of 15, 30, and 60 min were evaluated and since there was no difference in complement response between 30 and 60 min, 30 min was chosen. Another limitation of the study is that not all samples could be analyzed simultaneously. However, samples from both study groups were analyzed at each occasion and there was no statistically significant difference in bacterial load between groups. To further confirm that the methodological concept worked, we verified that the increase in complement activity that we noticed in both study groups after pneumococcal stimulation was statistically significant.

We cannot entirely exclude that the study was underpowered. The lack of previous research was of course one of the rationales for performing the study but naturally it hampered sample size calculations. What size a difference in complement activation should have to be clinically significant is not known. However, considering the strikingly similar response between the groups, it is doubtful whether a larger study would have resulted in a different outcome.

As a secondary finding, we noticed that the majority of the study participants, despite being unvaccinated, showed immunity to all the three investigated pneumococcal serotypes. This most likely supports studies showing that most individuals are exposed to and infected by pneumococci during childhood [23]. The antibody levels were equally distributed between individuals with and without CD so we found nothing that indicated differences in antibody response to pneumococci with regard to CD status.

In conclusion, this study tested a novel idea and even though no differences in complement response to pneumococci were found between CD patients and controls, this is an important pilot study which hopefully can be followed by further studies investigating involvement of complement as well as other immune mechanisms (e.g., IgG2 levels) that in addition to hyposplenism could contribute to the increased susceptibility to infectious complications in CD.

## Abbreviations

CD: Celiac disease
ELISA: Enzyme-linked immunosorbent assay
GFD: Gluten-free diet
HR: Hazard ratio
IPD: Invasive pneumococcal disease
MAC: Membrane attack complex
MBL: Mannan-binding lectin
tTG: Antibodies against tissue transglutaminase
